# No association of *GABRA1* rs2279020 and *GABRA6* rs3219151 polymorphisms with risk of epilepsy and antiepileptic drug responsiveness in Asian and Arabic populations: Evidence from a meta-analysis with trial sequential analysis

**DOI:** 10.3389/fneur.2022.996631

**Published:** 2022-09-14

**Authors:** Tiejun Zhang, Yi Yang, Xiutian Sima

**Affiliations:** ^1^Department of Neurosurgery, West China School of Medicine/West China Hospital, Sichuan University, Chengdu, China; ^2^Chengdu Seventh People's Hospital, Chengdu, China

**Keywords:** epilepsy, *GABRA1*, *GABRA6*, polymorphism, meta-analysis, trial sequential analysis (TSA)

## Abstract

The γ-aminobutyric acid type A receptors (GABA_A_R) have been reported to contribute to the pathogenesis of epilepsy and the recurrence of chronic seizures. Genetic polymorphisms in *GABRA1* and *GABRA6* may confer a high risk of epilepsy and multiple drug resistance, but with conflicting results. We aimed to assess the association of *GABRA1* rs2279020 and *GABRA6* rs3219151 with epilepsy risk using a meta-analysis. The databases of Pubmed, Ovid, Web of Science, and China National Knowledge Infrastructure were searched. Summary odds ratios (ORs) and 95% confidence intervals (CIs) were computed to evaluate the association between the polymorphisms and epilepsy risk using a fixed- or random-effect model. Trial sequential analysis (TSA) was performed to assess the results of the meta-analysis. No significant association between the *GABRA1* rs2279020 and *GABRA6* rs3219151 and the risk of epilepsy was found in the Asian and Arabic populations. The negative results were also observed when comparing the *GABRA1* rs2279020 and *GABRA6* rs3219151 polymorphism to antiepileptic drug responsiveness. The trial sequential analysis confirmed the results of the meta-analysis. This meta-analysis suggests that *GABRA1* rs2279020 and *GABRA6* rs3219151 are not risk factors for the etiology of epilepsy and antiepileptic drug responsiveness in the Asian and Arabic populations.

## Introduction

Among all neurological diseases, epilepsy is the most common one with over 50 million people diagnosed worldwide (1, 2). It is estimated that about 80% of the patients reside in resource-poor countries, causing an enormous medical, social, and economic burden (2). Although the etiology of epilepsy remains unclear, the amount of evidence has shown that genetic factors may contribute to the occurrence of epilepsy (3–5). Matuja et al. reported that family history was an independent factor for the risk of developing epilepsy, with an odd ratio of 3.52 (3). Kjeldsen et al. reported that monozygotic twins had a 5.4-fold higher risk of febrile seizures compared with dizygotic twins (4).

Antiepileptic drugs (AEDs) were commonly used to prevent epileptic seizures. Almost one-third of patients, however, continue to show recurrent seizures despite optimal AEDs treatment (6). Genetic abnormalities may disrupt pharmacokinetics and pharmacodynamics of AEDs, and thus affect treatment efficacy. As major targets of AEDs, γ-aminobutyric acid type A receptors (GABA_A_R) play pivotal roles in the suppression of epileptogenesis by maintaining homeostasis over brain excitation (7–10). In a rat model of temporal lobe epilepsy, the alteration in the structure and function of GABA_A_R subtypes may be involved in drug resistance (9, 10).

GABA_A_Rs are heteropentamers encoded by 19 subunits of α (1–6), β (1–3), γ (1–3), δ, ε, θ, π, and ρ (1–3) (11). The α1β2γ2 subunit is most abundant in almost all regions of the brain and usually underlies excitability disorders, such as epilepsy (7, 8). In human and animal studies, the differential expression and composition of GABA_A_Rs subunits may contribute to the pathogenesis of epilepsy and the recurrence of chronic seizures (12). Single nucleotide polymorphisms (SNP) in any of the subunits may alter the expression of GABA, and ultimately influence epilepsy seizures and responsiveness to AEDs (13–15). For example, Kumari et al. reported that the rs2279020 G allele in *GABRA1* conferred a high risk of epilepsy and multiple drug resistance (16), whereas Al-Eitan et al. reported that the rs2279020 polymorphism did not show any linkage with the occurrence of epilepsy and treatment responsiveness (17). Given the conflicting results, a meta-analysis is of great importance to be performed to obtain the real effect of the rs2279020 on epilepsy risk. Moreover, the relationship between rs3219151 polymorphism in *GABRA6* and the susceptibility of epilepsy is also evaluated in this meta-analysis. We found that neither the *GABRA1* rs2279020 nor the *GABRA6* rs3219151 polymorphism was a risk factor for the etiology of epilepsy and antiepileptic drug responsiveness.

## Materials and methods

### Search strategy

The methodology of this meta-analysis followed the PRISMA 2020 statement guidelines. We identified records from the databases of Pubmed, Ovid, Web of Science, and the China National Knowledge Infrastructure using the following searching strategies: (gamma-aminobutyric acid type A receptor subunit alpha1 or *GABRA1* or gamma-aminobutyric acid type A receptor subunit alpha6 or *GABRA6*) and (polymorphis^*^ or variant^*^ or SNP^*^) and (epilepsy). The literature screening was performed until 18 May 2022 without language and ethnic restrictions. If the language of publication was non-English, we translated the required information into English.

### Inclusion and exclusion criteria

The inclusion criteria were as follows: (a) investigated the association of *GABRA1* rs2279020 and *GABRA6* rs3219151 polymorphisms with the risk of epilepsy or outcome of AED treatment; (b) contained sufficient original data to compute summary odds ratios (ORs) and 95% confidence intervals (CIs). The exclusion criteria were as follows: (a) review articles; (b) absence of available data; and (c) overlapping data reported by the same research group.

### Data extraction

ZT and YY independently extracted the following data from the included studies: the name of the first author, year of publication, ethnicity of study population, number of cases and controls, criteria used to define drug-responsiveness and drug-resistance, and genotyping technique and genotype distributions of *GABRA1* rs2279020 and *GABRA6* rs3219151 polymorphisms.

### Assessment of study quality

ZT and YY independently assessed the eligible studies using the modified Newcastle-Ottawa Scale (18). Studies were graded 0–8 stars based on the following items: the selection of the study population, the comparability of cases and controls, and the ascertainment of the outcome. Studies with equal to or more than four stars were considered high quality and studies with <4 stars were considered low quality. Any disagreement during the assessment was resolved by discussion with SX.

### Trial sequential analysis

During the comparison of the *GABRA1* rs2279020 and *GABRA6* rs3219151 polymorphisms with the risk of epilepsy and drug resistance, TSA software (version 0.9.5.10 beta) was performed to evaluate the reliability of the meta-analysis under a recessive model. The O'Brien-Fleming boundary, sequential monitoring boundary, futility boundary, and cumulative test statistic (Z-curve) were calculated with an alpha of 5%, power level of 80%, and relative risk reduction of 20% (19).

### Statistical analysis

Data analysis was carried out using the STATA software version 10 (STATA Corporation, College Station, TX). The pooled odds ratio (OR) and its 95% confidence interval (CI) were calculated to assess the association of the *GABRA1* rs2279020 and *GABRA6* rs3219151 polymorphisms with the risk of epilepsy and AEDs responsiveness. Stratified analyses were also performed if two or more studies were available based on ethnicity (Asian and Arabian), the Hardy–Weinberg equilibrium (HWE), and study quality (high and low). Cochran's *Q*-test and *I*^2^ statistics were used to assess the inter-study heterogeneity (20). A significant *Q* statistic (*P* < 0.10) indicated high heterogeneity. Therefore, a random-effects model was used for the meta-analysis (21) and meta-regression was used to explain the origin of the heterogeneity. Otherwise, a *P*-value of more than 0.10 indicated low heterogeneity, and then a fixed-effect model was applied (22). Sensitivity analysis was performed to estimate the effect when an individual study was sequentially excluded from the summary analysis. According to the guideline of meta-analysis, a publication bias may be done if eligible studies were more than nine. In this meta-analysis, the highest number of studies in all comparisons was eight. Publication bias, therefore, was not performed.

## Results

### Flow diagram of selection studies

**Figure 1:**
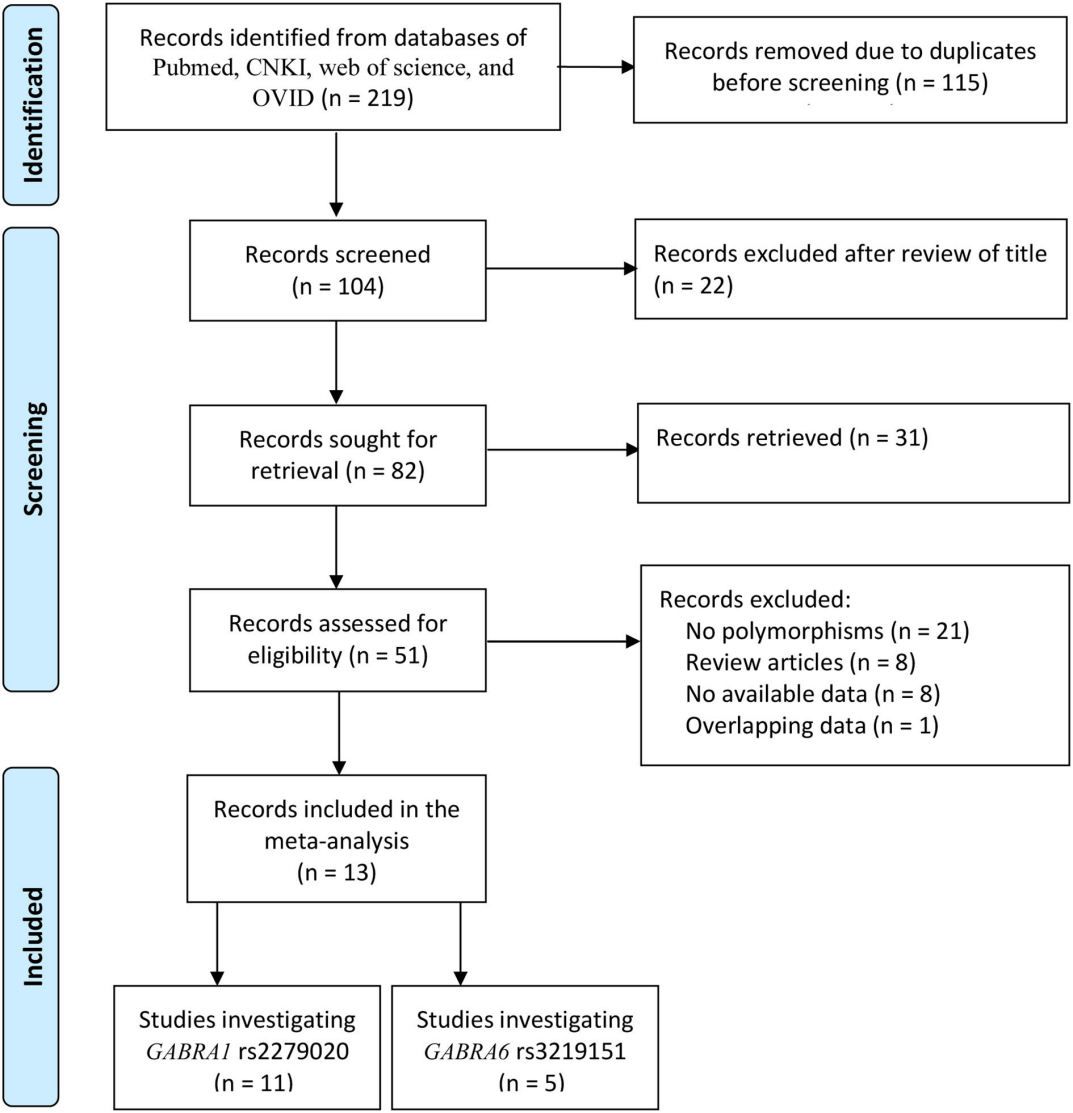
Flow diagram of selection studies.

In total, 219 records were identified after database searching. Before the screening, 115 records were removed due to duplicates. The remaining 104 records were sought for retrieval, and 53 were excluded after reviewing the title and the abstract. Full texts of the remaining 51 records were assessed for eligibility, and 38 were excluded due to no *GABRA1* rs2279020 and *GABRA6* rs3219151 polymorphisms (*n* = 21), lack of available data (*n* = 8), review articles (*n* = 8), and overlapping data (*n* = 1). Finally, 13 studies were included in this meta-analysis, including 11 investigating *GABRA1* rs2279020 polymorphism and five investigating *GABRA6* rs3219151 polymorphism (Figure 1).

### Characteristics of included studies

The characteristics of the included studies are summarized in Table 1. Of the 13 studies, 11 were carried out in Asians and 2 in Arabians. Various genotyping assays for analyzing the *GABRA1* rs2279020 and *GABRA6* rs3219151 polymorphisms were reported, namely, polymerase chain reaction-restriction fragment length polymorphism, matrix-assisted laser desorption/ionization time-of-flight mass spectrometry, TaqMan, Illumina VeraCode GoldenGate genotyping assay, SnapShot, and Sanger sequencing. Approximately half of the included studies described matched case-control factors, such as age, gender, ethnicity, and residence. However, <30% (4/13) of the studies used the second genotyping method for quality control in this study.

**Table 1:** Characteristics of the reports included in the meta-analysis.

**References**	**Year**	**Country**	**Ethnicity**	**Number of cases and controls**	**Definition**		**Matching criteria**	**Genotyping method**	**Quality control**	**New-Castle Ottawa scale**	**Polymorphisms**
					**NR**	**R**					
Abdalla et al. (23)	2021	Saudi Arabia	Arabian	23/35	NA	NA	Age, gender, ethnicity, and residence	PCR-RFLP	NA	⋆⋆⋆⋆	rs2279020
Al-Eitan et al. (17)	2020	Jordan	Arabian	296/299	Failure of adequate trials of AED treatments	Seizure-free for at least three times in the previous 12 months	NA	MassARRAY	NA	⋆⋆⋆	rs2279020
Amjad et al. (13)	2022	Pakistan	Asian	80/100	NA	NA	Age and gender	PCR-RFLP	Regenotyping	⋆⋆⋆⋆	rs2279020
Baghel et al. (24)	2016	India	Asian	478/170	At least one seizure in the previous 10 months following AED treatments	Freedom from seizures in the previous 10 months	Ethnicity	MALDI-TOF	Regenotyping	⋆⋆⋆⋆	rs2279020 and rs3219151
Balan et al. (25)	2013	India	Asian	441/267	At least 12 seizures per year for more than 2 years after monotherapy and duotherapy trials	Freedom from seizures for at least 1 year with AED therapy	Age and gender	Taqman	NA	⋆⋆⋆⋆	rs2279020 and rs3219151
Bhat et al. (26)	2018	India	Asian	100/100	NA	NA	Age and gender	PCR-RFLP	NA	⋆⋆⋆⋆	rs2279020
Cao and Xiao (27)	2013	China	Asian	480 patients	Failure of adequate trials of AED treatments	Freedom from seizures for at least 12 months	NA	Illumina VeraCode GoldenGate genotpying assay	NA	⋆⋆⋆	rs2279020
Feng et al. (28)	2016	China	Asian	111 pediatric patients	Seizure frequency of ≤ 50% reduction within 1 year	Seizure frequency of >50% reduction within 1 year	NA	MassARRAY	Regenotyping	⋆⋆⋆	rs2279020
Kumari et al. (16)	2010	India	Asian	395/199	At least four seizures over a period of 1 year with three AED treatments	Freedom from seizures for at least 1 year	NA	PCR-RFLP	NA	⋆⋆⋆	rs2279020
Kumari et al. (29)	2011	India	Asian	401/202	At least four seizures over a period of 1 year with three AED treatments	Freedom from seizures for at least 1 year	NA	PCR-RFLP	Regenotyping	⋆⋆⋆	rs3219151
Pan and Wang (30)	2014	China	Asian	205/83	NA	NA	NA	SnapShot	NA	⋆⋆⋆	rs2279020
Prasad et al. (31)	2014	India	Asian	310/310	NA	NA	Age and gender	PCR-RFLP	NA	⋆⋆⋆⋆	rs3219151
Riaz et al. (32)	2021	Pakistan	Asian	150/150	NA	NA	Ethnicity and geographic area	Sequencing	NA	⋆⋆⋆⋆	rs2279020 and rs3219151

NR, drug resistant; R, drug responsive; NA, not available; PCR-RFLP, polymerase chain reaction-restriction fragment length polymorphism; AED, anti-epileptic drugs; MALDI-TOF, matrix-assisted laser desorption/ionization time-of-flight. The stars indicate the score of NEW-Castle Ottawa Scale.

### Meta-analysis of the association of the *GABRA1* rs2279020 and *GABRA6* rs3219151 polymorphisms with risk of epilepsy in Asian and Arabic populations

**Figure 2:**
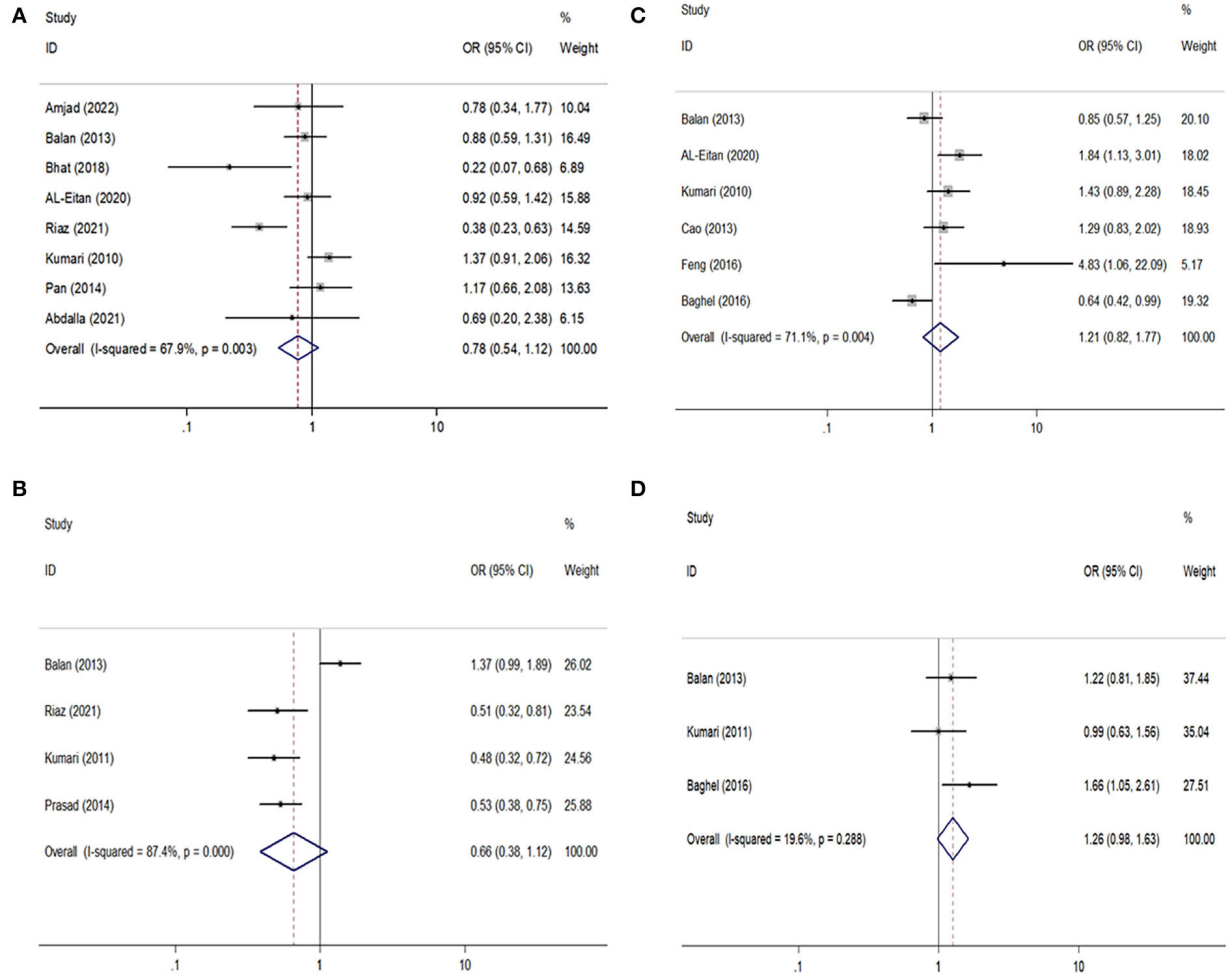
Forest plots of *GABRA1* rs2279020 and *GABRA6* rs3219151 polymorphisms with the risk of epilepsy and drug resistance. **(A,B)** Comparison of rs2279020 and rs3219151 with the risk of epilepsy; **(C,D)** comparison of rs2279020 and rs3219151 with the risk of drug resistance.

For the *GABRA1* rs2279020 polymorphism, eight studies were included involving 1,688 patients with epilepsy and 1,233 controls. Among them, 75% (6/8) were performed in Asians and 25% (2/8) were performed in Arabians; 62.5% (5/8) were high-quality studies and did not derive from HWE in controls. In an overall analysis, no significant association between the *GABRA1* rs2279020 and the risk of epilepsy was found. Similarly, the negative results were also observed in subgroup analyses based on ethnicity and study quality. Even though the *GABRA1* rs2279020 was associated with an increased risk of epilepsy among studies without HWE under heterozygous comparison (OR = 1.83, 95% CI, 1.30–2.58) and dominant genetic model (OR = 1.52, 95% CI, 1.13–2.03), the polymorphism was not associated with the risk of epilepsy among studies with HWE in controls (Table 2, Figure 2A). Therefore, we may conclude that the *GABRA1* rs2279020 was not a risk factor for the occurrence of epilepsy.

**Table 2:** Meta-analysis of *GABRA1* rs2279020 and *GABRA6* rs3219151 polymorphisms with the risk of epilepsy.

**Polymorphisms**	**Variables**	***n***	**Heterozygous comparison**		**Homozygous comparison**		**Dominant model**		**Recessive model**	
			**OR (95% CI)**	***P^*a*^***	**OR (95% CI)**	***P^*a*^***	**OR (95% CI)**	***P^*a*^***	**OR (95% CI)**	***P^*a*^***
rs2279020	Total	8	1.18 (0.98–1.41)	0.12	0.93 (0.64–1.37)	0.04	1.12 (0.94–1.32)	0.15	0.78 (0.54–1.12)	0.003
	Ethnicity									
	Asian	6	1.23 (0.84–1.80)	0.04	0.90 (0.52–1.54)	0.01	1.10 (0.79–1.54)	0.05	0.74 (0.46–1.20)	0.001
	Arabian	2	1.09 (0.77–1.54)	0.80	0.94 (0.59–1.48)	0.78	1.05 (0.76–1.44)	0.99	0.89 (0.59–1.34)	0.68
	HWE									
	Yes	5	0.98 (0.79–1.22)	0.93	0.91 (0.68–1.20)	0.92	0.96 (0.78–1.18)	0.94	0.78 (0.53–1.13)	0.03
	No	3	1.83 (1.30–2.58)	0.65	0.76 (0.21–2.75)	0.01	1.52 (1.13–2.03)	0.24	0.65 (0.21–2.03)	0.01
	Study quality									
	High	5	1.06 (0.81–1.40)	0.22	0.71 (0.50–1.01)	0.28	0.93 (0.72–1.20)	0.99	0.56 (0.34–0.93)	0.04
	Low	3	1.24 (0.83–1.85)	0.09	1.30 (0.97–1.75)	0.15	1.25 (0.83–1.88)	0.06	1.15 (0.88–1.49)	0.42
rs3219151	Total	4	0.72 (0.46–1.15)	0.001	0.51 (0.18–1.42)	<0.001	0.66 (0.38–1.12)	<0.001	0.59 (0.30–1.17)	<0.001
	Study quality									
	High	3	0.78 (0.44–1.40)	0.001	0.62 (0.13–2.97)	<0.001	0.73 (0.37–1.42)	<0.001	0.64 (0.20–2.01)	<0.001

^a^P_−_value for heterogeneity test; OR, odds ratio; CI, confidence interval; HWE, Hardy-Weinberg equilibrium.

For the *GABRA6* rs3219151 polymorphism, four studies were included involving 1,299 patients with epilepsy and 929 controls. All the studies were performed in Asians and were in HWE except for one (32). No significant association between the *GABRA6* rs3219151 and the risk of epilepsy was found in both overall comparisons and subgroup analyses based on study quality (Table 2,Figure 2B).

### Meta-analysis of the association of the *GABRA1* rs2279020 and *GABRA6* rs3219151 polymorphisms with risk of drug resistance in Asian and Arabic populations

For the *GABRA1* rs2279020 polymorphism, six studies were included involving 1,860 patients. High heterogeneity among studies was observed in overall analysis rather than in subgroup analysis based on study quality. The meta-regression analysis also showed that study quality can explain all the heterogeneity under heterozygous comparison and dominant genetic model. There was no significant association of the *GABRA1* rs2279020 polymorphism with resistance to AEDs in the overall pooled populations and Asians. As a matter of interest, a significant association of the *GABRA1* rs2279020 polymorphism with resistance to AEDs was observed in low-quality studies (heterozygous comparison: OR = 1.49, 95% CI, 1.12–1.98; dominant model: OR = 1.57, 95% CI, 1.20–2.04; recessive model: OR = 1.31, 95% CI, 1.01–1.71) but not in the high-quality studies (Table 3, Figure 2C), indicating that the negative results may be more reliable.

**Table 3:** Meta-analysis of *GABRA1* rs2279020 and *GABRA6* rs3219151polymorphisms with drug-resistance epilepsy.

**Polymorphisms**	**Variables**	***n***	**Heterozygous comparison**		**Homozygous comparison**		**Dominant model**		**Recessive model**	
			**OR (95% CI)**	***P^*a*^***	**OR (95% CI)**	***P^*a*^***	**OR (95% CI)**	***P^*a*^***	**OR (95% CI)**	***P^*a*^***
rs2279020	Total	6	1.15 (0.79–1.67)	0.01	1.30 (0.83–2.02)	0.02	1.21 (0.82–1.77)	0.004	1.17 (0.94–1.45)	0.18
	Ethnicity									
	Asian	5	1.00 (0.70–1.43)	0.08	1.26 (0.75–2.14)	0.01	1.09 (0.73–1.63)	0.02	1.18 (0.94–1.48)	0.11
	Study quality									
	High	2	0.74 (0.54–1.00)	0.62	0.78 (0.52–1.17)	0.15	0.75 (0.56–1.00)	0.36	0.93 (0.64–1.34)	0.17
	Low	4	1.49 (1.12–1.98)	0.31	1.24 (0.97–1.60)	0.29	1.57 (1.20–2.04)	0.34	1.31 (1.01–1.71)	0.32
rs3219151	Total	3	1.32 (1.01–1.72)	0.30	1.13 (0.81–1.58)	0.58	1.26 (0.98–1.63)	0.29	0.95 (0.71–1.27)	0.93

For the *GABRA6* rs3219151 polymorphism, three studies were included involving 1,202 patients. A borderline significant association of the *GABRA6* rs3219151 polymorphism with resistance to AEDs was detected under a heterozygous comparison (OR = 1.32, 95% CI, 1.01–1.72) (Table 3, Figure 2D).

### Sensitivity analysis

A reverse outcome of the *GABRA1* rs2279020 polymorphism with epilepsy risk was observed when the study by Balan et al. was excluded from the heterozygous comparison (25) and the study by Kumari et al. from the recessive genetic model (16). The reverse outcome of the *GABRA1* rs2279020 polymorphism with resistance to AEDs was also observed when the study by Baghel et al. was excluded (24).

### TSA analysis

**Figure 3:**
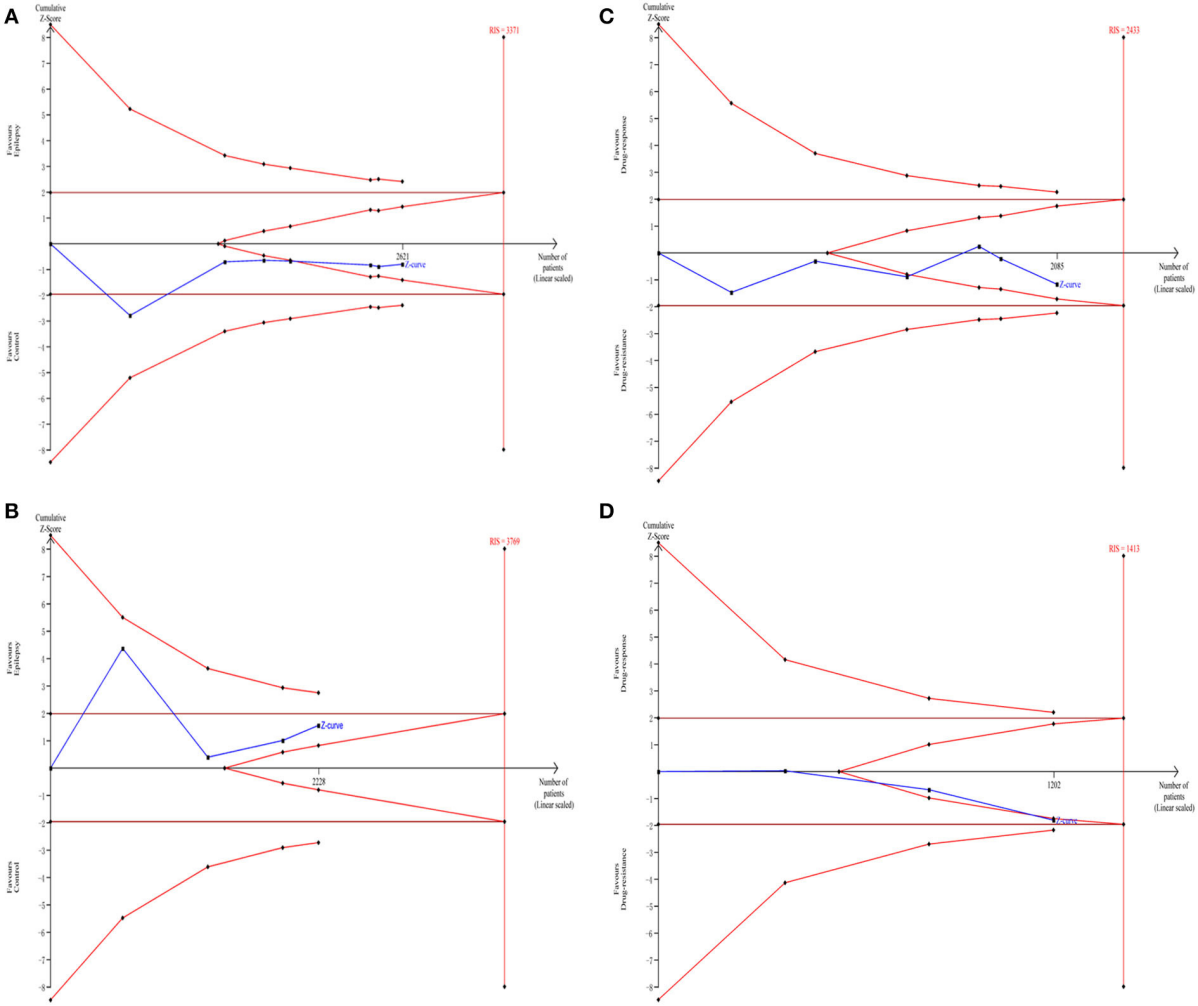
Trial sequential analysis of the association between rs2279020 and rs3219151 and the risk of epilepsy **(A,B)**. Trial sequential analysis of the association between rs2279020 and rs3219151 and the risk of drug resistance **(C,D)**. RIS, required information size.

The TSA analysis was used to evaluate the reliability of the meta-analysis. As shown in Figures 3A,C,D, the cumulative Z-curve crossed the futility boundary, supporting the negative results in the meta-analysis. However, the cumulative Z-curve in Figure 3B did not cross any boundary, indicating that additional studies investigating the association between the *GABRA6* rs3219151 polymorphism and epilepsy risk are required.

## Discussion

The GABA, a crucial inhibitory neurotransmitter, has a role in both pre- and post-synaptic neuronal processes by binding to GABA_A_Rs. Genetic mutations in *GABA*_*A*_*R* subunits have been demonstrated to be the risk factors for the development of epilepsy through multiple mechanisms, including reduction of mRNA stability, impairment of subunit folding, and aberrant glycosylation causing inhibition of receptor assembly and trafficking (33). In addition, *GABA*_*A*_*R*s constitute the targets for many AEDs such as benzodiazepines, phenobarbital, gabapentin, and topiramate (34). Therefore, the SNPs in *GABA*_*A*_*R* subunits may influence the susceptibility of epilepsy and different responsiveness to AEDs.

Previously, an SNP located in intron 10 of *GABRA1* (rs2279020) was reported to be potentially functional, with the A → G shift changing the shape of the protein that led to alternative splicing (32). A significant association of the rs2279020 with epilepsy risk was reported by some research groups (16, 26, 32), whereas no significant association was reported by other research groups (13, 17, 23, 25). The controversial results were also observed in comparison to the rs2279020 with AED resistance (16, 17, 24, 25). For example, some authors reported that the rs2279020 was a risk factor for affecting treatment efficacy (16, 17). However, some authors reported that the rs2279020 was not associated with AED resistance (24, 25). One reason for the inclusive results may be the insufficient power due to small samples in each individual study. The meta-analysis was then used to overcome the drawback by pooling all the published data together. The findings from the current meta-analysis showed no correlation of the rs2279020 to the susceptibility of epilepsy and AED responsiveness. Furthermore, the TSA revealed that the cumulative Z-curve crossed the futility boundary, indicating that the negative results may be robust.

The rs3219151, located in the 3′ untranslated region of *GABRA6* was predicted to affect the microRNA-binding site activity (35). The association of the rs3219151 with epilepsy susceptibility and AED resistance was also examined in a series of studies, but with uncertain findings. Balan et al. reported that the rs3219151 was not related to epilepsy susceptibility and AED resistance (25). However, Riaz et al. (32), Kumari et al. (29), and Prasad et al. (31) reported that the rs3219151 was a significant risk factor for developing epilepsy. By pooling all the original data together, this meta-analysis found no association of the rs3219151 with epilepsy susceptibility but a borderline significant association with AED resistance. The results seem to be unreliable for the following reasons. First, subsequent TSA showed that the current evidence was insufficient. Second, each technique has an advantage and a risk for genotyping error. To make the genotyping results robust, it is necessary to perform quality control by using a Sanger sequencing confirmation, which is a gold standard for genotyping (36). However, <30% of the studies used the second genotyping method for quality control in this study. Finally, there were only four studies investigating the distribution of the rs3219151 among cases and controls and only three studies investigating the difference of the rs3219151 among patients with AED responsiveness and resistance, which may make the findings uncertain. Further association studies, therefore, should be performed to confirm the results in the meta-analysis.

Heterogeneity analysis in this study showed that strong heterogeneity was observed in some overall comparisons. The meta-regression analysis was used to explore the possible reason, and we found that study quality can explain all the heterogeneity, suggesting that high-quality studies were so important to obtain precise observational data. The sensitivity analysis revealed that the outcome altered if we excluded a single study each time (16, 24, 25). Only studies carried out with the Asian and Arabic populations were included in this study. As it is necessary for association studies performed in diverse ethnic groups, additional studies involving subjects of different races are required. There are different types of epilepsy, and thus it is of great value to perform subgroup analysis based on the subtype of epilepsy. However, insufficient data in this study prevented our further subgroup analysis. Although the limitations existed in this meta-analysis, the evidence of null association of the rs2279020 with epilepsy risk and AEDs resistance seems to be reliable based on supporting data from TSA. However, further studies evaluating the effect of rs3219151 on the predisposition of epilepsy are of great importance, especially in Africans and Caucasians.

In conclusion, the present accumulated evidence revealed that neither the *GABRA1* rs2279020 nor the *GABRA6* rs3219151 polymorphism was a risk factor for the etiology of epilepsy and antiepileptic drug responsiveness in the Asian and Arabic populations. Larger population-based case-cohort studies are warranted to verify the quantitative association in diverse ethnicities, especially in Africans and Caucasians.

## Data availability statement

The original contributions presented in the study are included in the article/supplementary material, further inquiries can be directed to the corresponding author/s.

## Author contributions

XS designed and wrote the manuscript. TZ and YY collected and analyzed the data. All authors contributed to the article and approved the submitted version.

## Funding

This work was supported by grants from the 1.3.5 project for disciplines of excellence, the West China Hospital, Sichuan University (Grant Nos. TJZ202006 and 20HXJS007), the Key Project for Science and Technology Department of Sichuan Province (Grant No. 2018SZ0216), and the Sichuan University (Grant No. 2017scu11038).

## Conflict of interest

The authors declare that the research was conducted in the absence of any commercial or financial relationships that could be construed as a potential conflict of interest.

## Publisher's note

All claims expressed in this article are solely those of the authors and do not necessarily represent those of their affiliated organizations, or those of the publisher, the editors and the reviewers. Any product that may be evaluated in this article, or claim that may be made by its manufacturer, is not guaranteed or endorsed by the publisher.
